# Time-related changes in quality of life in persons with lower limb amputation or spinal cord injury: protocol for a systematic review

**DOI:** 10.1186/s13643-019-1108-3

**Published:** 2019-08-02

**Authors:** Matthew Young, Carly McKay, Sean Williams, Peter Rouse, James L. J. Bilzon

**Affiliations:** Department for Health, University of Bath, Bath, BA2 7AY UK

**Keywords:** Systematic review, Lower limb amputation, Spinal cord injury, Quality of life, SF-36, WHOQOL

## Abstract

**Background:**

Experiencing a lower limb amputation (LLA) or spinal cord injury (SCI) is a life-changing event, affecting physical and systemic function as well as having psychological and social impacts. However, the severity of the physical impairment and/or motor disability demonstrates a poor relationship with patient-reported quality of life, suggesting that other factors determine such outcomes. As such, holistic health-related quality of life (QoL) assessment is an important tool to monitor long-term outcomes. While there are some studies that have assessed the influence of variables such as age at time of injury occurrence and time since injury on changes in QoL, there are no systematic reviews which synthesise this evidence.

**Methods/design:**

All follow-up study designs will be included, where data from multiple time points are presented. Searches will target both SCI and LLA populations where a validated measure of QoL has been used: Medical Outcome Study Short-Form 36/12 or the World Health Organization Quality of Life instruments 100 and BREF. Studies must include adult participants (≥ 18 years at time of injury) and detail time since injury event and patient age.

The primary objective is to establish the effects of participant age and time since injury on QoL scores. Secondary objectives include determining between-group effects (i.e. LLA vs. SCI).

We will search PubMed, Embase and Web of Science databases, supplemented by hand-searching references within existing review articles and experimental studies. Reviewer pairs will conduct screening and quality assessment of included papers.

Results will be stratified by impairment, QoL tool, age/time since injury and additional variables such as sex, race, comorbidity or disease aetiology, as appropriate. If sufficient high-quality data exist, a meta-analysis will be conducted.

**Discussion:**

The results of this systematic review will summarise evidence of how QoL changes across the life course, relative to both patient age and time since injury, for both LLA and SCI populations. By enabling a direct comparison of different chronic conditions, disability-specific differences in QoL changes over the life course can be identified.

**Systematic review registration:**

PROSPERO CRD42018096633.

**Electronic supplementary material:**

The online version of this article (10.1186/s13643-019-1108-3) contains supplementary material, which is available to authorized users.

## Background

Sustaining a lower limb amputation (LLA) or spinal cord injury (SCI) is a significant event, with permanent implications for the individual’s future life. The outcomes of both LLA and SCI are highly individualised and heterogeneous, in part due to variability in the level of injury/amputation and, at least for SCI, the degree of injury completeness. The reasons for injury occurrence can also be important, with many LLA cases being related to significant existing chronic disease and related comorbidities. However, both conditions present a series of shared outcomes that have a significant impact on aspects of quality of life (QoL) [1]. For example, mobility restriction and differences in appearance lead to shared challenges around social engagement, perceived isolation and dependency, which can limit community participation [2–7] and increase risk of depression [8–11]. Similarly, a general loss of skeletal muscle mass and restriction of physical activity contribute to long-term health consequences associated with early ageing [12, 13] such as increased risk of cardiovascular and metabolic diseases [14–16].

Notwithstanding, several studies have demonstrated that for individuals with a chronic motor disability the level of physical impairment does not directly correlate with patient-reported quality of life [17–20]. QoL scales consider the resultant limitations and consequences of a disability and assess how these change over time, even when the underlying injury remains. As such, QoL measures are increasingly being recognised as an important tool for monitoring long-term outcomes following rehabilitation [21–24]. Used in combination with assessment of functional performance, they assist in the recording of changing perceptions and patient adjustment, as well as their acceptance of life post-injury [25, 26].

Researchers in both LLA and SCI communities acknowledge the value of QoL measures as longitudinal assessment tools [17, 27–31]. They provide a quantitative mechanism for capturing changes in post-injury health and broad level treatment outcomes, both for an individual and for a disability group, relative to population normative data. However, there is a lack of homogenous outcome data for both communities and a lack of consensus around QoL scale selection.

In part, this is due to the multifaceted nature of QoL as a construct. There are varying definitions and tools available, built around both conceptual understandings of what constitutes a person’s quality of life and design considerations specifying what facets a tool is designed to assess and for which populations. One widely utilised distinction is that between ‘objective’ and ‘subjective’ assessment measures, previously described by Dijkers and Fuhrer [25, 26]. Several LLA and SCI reviews have followed this construct and divided existing QoL measures under these headings [27–29, 32]. Within this distinction, objective measures consider external definitions of QoL (i.e. those that are societally or culturally defined), whereas subjective measures focus on an individual’s perceptual assessment of their life relative to their expectations.

These distinctions are particularly important to consider when QoL tools have been deployed longitudinally, or when trying to capture within-population differences in response to rehabilitation or treatment. Both components of QoL should be reflected when attempting to create more personalised rehabilitation or recovery pathways for specific patient sub-groups. For example, there are ongoing shifts in the population demographics for both SCI and LLA [33–35]. For SCI, age at onset is subject to considerable variation globally, with increasingly ageing populations in more economically developed countries (MEDC) highlighted as a potential future public health challenge [36]. Similarly for LLA, there is significant variation between countries for age at onset, aetiology and incidence [37, 38]. This variation encompasses a complex landscape, with regional public health provision intersecting with clinical advances.

Regardless, within both conditions, mean and upper age at onset and potentially life expectancy is increasing for both populations, in particular within MEDCs [34, 35, 39–41]. Furthermore, it has been noted that a bimodal distribution may be forming for both conditions, [35, 42] with traumatic injuries relating to misadventure or conflict continuing to occur at younger ages, while the proportion whose injury results from age- or lifestyle-related factors, such as vascular disease for LLA or frailty-related falls for SCI, increases.

This potentially growing divide in age at time of injury occurrence raises questions around identifying rehabilitation or treatment requirements for different age groups [43, 44]. It also has implications when considering functional and QoL outcomes across the life course and when selecting QoL instruments [45–47].

Previous research has been undertaken comparing SCI and LLA population QoL outcomes to population norms and healthy controls [27], but no cross-population comparisons have been reported. This leads to important questions regarding how the comparative success of long-term rehabilitation and recovery pathways can be evaluated and optimised to sustain QoL. By directly comparing the QoL scores at an individual health domain level (e.g. Physical Health, Psychological, Social Relationship and Environment for WHOQOL-BREF), we may gain insight into the condition-specific challenges of living with these disabilities.

There is a significant challenge in assessing long-term quality of life changes relative to age, due in part to a lack of longitudinal data in both SCI and LLA populations, particularly for those who suffered an injury in later life [19, 34, 48, 49]. As population demographics change and post-operative life expectancy rates can be expected to improve, the detail of variation in QoL relative to time since injury and age at onset holds increasing importance [33, 45]. There is currently a lack of consensus around the relationship between QoL with age at time of injury and time since injury, both for persons with SCI [48] and those with LLA [50, 51].

This review intends to provide a synthesis of these previously unassessed variables, alongside aetiology characteristics, to address this issue. This is required as the emergence of increasingly divided points of disability incidence may necessitate changes in care provision or categorisation.

### Aim

The primary purpose of this systematic review is to evaluate current evidence to determine the independent and combined effects of age at time of injury and time since injury on health-related quality of life in persons with spinal cord injury or lower limb amputation. Where there are a sufficient number and quality of articles, analyses will be performed to determine differences in the effects of these variables, between groups and over time. The secondary purpose of the review is to determine the potential effects of other variables (e.g. sex, ethnicity, injury type impairment/function score).

## Methods/design

This protocol follows the principles of The Cochrane Handbook for Systematic Reviews, and the final report will conform to the Preferred Reporting Items for Systematic Reviews and Meta-Analyses [52, 53]. The Preferred Reporting Items for Systematic Reviews and Meta-Analyses (PRISMA-P) process will be followed, as presented in Additional file 1.

This protocol was registered with the PROSPERO International Prospective Register of Systematic Reviews database (registration number CRD42018096633).

### QoL tool selection

When deciding which quality of life (QoL) measures to include in this review, we required scales which (i) had been assessed for validity and reliability in both SCI and LLA populations, (ii) were in common use and, if possible, (iii) reflected both aspects of subjective and objective QoL assessment [25].

Several reviews have summarised both the utilisation and validity of QoL tools for SCI and LLA populations [27–31, 48, 54, 55]. These indicate that the WHOQOL-BREF [56] and Medical Outcomes SF-36 [57] are in common use and considered amongst the best available generic tools for both groups. The SF-36 is an objective tool, and the WHOQOL-BREF is considered subjective, although it has objective elements [27, 32, 54].

The SF-36 has been reported as one of the most widely evaluated generic health measures [58], and this is consistent with utilisation in LLA and SCI populations [48, 55, 59]. The SF-12 is also used in both populations, retains the same sub-domains as the SF-36 and has been shown to have good agreement with the longer form [60]. While the WHOQOL-BREF has been used less frequently, particularly in studies of persons with LLA, it has been extensively assessed [24, 32, 61–63] and is sometimes preferred because of its mixed subjective/objective features [29].

These scales are both subdivided into domains allowing sub-component analysis. Furthermore, the convergence and agreement between the two scales has been previously assessed and found acceptable in SCI and 27 other health conditions, although LLA has not been investigated as an independent health condition [32, 61]. For completeness, although it is used less frequently, data derived from the full WHOQOL-100 [64] survey will also be included.

### Eligibility criteria for consideration of inclusion

We will adopt an inclusive approach where all follow-up study designs, where data from multiple time points are presented, with any publication date, written in the English Language, will be eligible if they present data on adults (≥ 18 years at time of injury) with either SCI or LLA.

Studies will be required to utilise either (or combinations of) the Medical Outcome Study Short-Form 36/12 or the World Health Organization Quality of Life instruments 100 and BREF to assess patient health-related quality of life and provide sufficient detail to extract domain level scores. That is, the WHOQOL instruments define 4 domains (i.e. Physical Health, Psychological, Social relationship and Environment), while the Medical Outcome Study details two domains, Physical and Mental, which have four subsections each.

Additionally, studies must detail the sample’s age and/or the time since injury event/onset and present data from at least two follow-up time points. Furthermore, only LLA studies examining above ankle amputation will be included.

### Information sources and literature search strategy

The literature search strategy will be based around the digital libraries of PubMed, Embase and Web of Science Core Collection. This will be supplemented through a hand search of references within existing review articles and selected experimental studies.

Specific search strategies will utilise both medical subject headings (MeSH) or database-specific equivalents and free text terms to maximise study identification. These are detailed in Additional file 2.

### Study selection process

The citations of all records retrieved with be downloaded to Mendeley Desktop [65] (Version 1.17.13) for record keeping and duplicate removal. The title and abstract of entries will then be transferred to a Microsoft Excel [66] spreadsheet for appraisal and selection for full paper review. To minimise the risk of investigator bias, a team of four independent reviewers will be divided into two reviewer pairs to conduct all levels of screening and quality assessment. Use of multiple reviewer pairs will allow the rotation of citation assessment at abstract and full paper stages to further reduce reviewer bias. Discrepancies will be resolved by within-pair discussion and then by inclusion of an additional independent reviewer until consensus is reached. Cohen’s kappa statistic [67] will be calculated to determine the inter-rater agreement for study inclusion.

### Data extraction

Data to be extracted will include basic descriptive characteristics of the study (e.g. study design, year of publication, sample size, country of study); baseline participant characteristics both for injury type (LLA/SCI) and level of injury (i.e. above/below knee amputation; American Spinal Injury Association (ASIA) Impairment Scale and level of injury), aetiology of injury (i.e. trauma, vascular, cancer, etc.), personal characteristics (i.e. age, sex, comorbidity, time since injury, social status) and outcome results (i.e. quality of life scale used and scores across domains/sub-domains). The data extraction form will be pilot tested by two reviewers on 10 papers [68] and modified to reflect the level and specificity of data available.

### Data synthesis

The results of the systematic review will be summarised descriptively and quantitatively, assuming the appropriate data are available. Data will be considered across the two QoL scales, with results grouped by scale sub-domains, injury type and sub-population. We will conduct separate sub-group analyses across these strata and provide composite information where possible. Normative population data, where available, will also be referenced for comparative analysis. As age and time since injury are key variables, where these data are not available, additional data will be requested from corresponding authors.

Where sufficient data for meta-analysis are available, a mixed effects meta-regression model using the ‘metafor’ package in R (version 3.2.4, R Foundation for Statistical Computing, Vienna, Austria) will be used [69]. The modifying effects of age and time since injury on QoL outcomes will be evaluated as the change associated with a two standard deviation (SD) change in the predictor (i.e. a typically low vs. a typically high value) [70]. The random effects in the model will be a between-study SD, representing the typical difference in the true value of the effect in different study settings, plus a within-study random effect to account for within-study repeated measurements.

### Quality assessment

The risk of bias within individual studies will be assessed using the Cochrane Risk of Bias Tool [52] for randomised trials, and the NIH Quality Assessment Tool for Observational Cohort and Cross-Sectional Studies [71]. Regardless of quality score, papers will be included in the final review and analyses, but summarised descriptively according to risk of bias. Corresponding authors will be contacted if insufficient details exist to confidently assess the risk of bias in individual studies.

## Discussion

This systematic review and meta-analysis is designed to assess the independent and combined effects of age at time of injury and elapsed time since injury on quality of life in persons with chronic SCI and LLA.

By taking a cross-population perspective, this review will highlight potential discrepancies in QoL outcome for each group across the various sub-domains of the two QoL scales. This may also allow further comparative analysis between the SF-36 and WHOQOL BREF scales for use in disabled populations. Given the heterogeneity of aetiology for both conditions, a comparative view of rehabilitative outcomes is necessary to identify any global failings or successes of rehabilitative practice for the different conditions.

## Additional files

Additional file 1: PRISMA-P 2015 checklist. (DOCX 38 kb)

Additional file 2: Search terms. (DOCX 76 kb)

## Data Availability

The datasets generated and/or analysed during the current study are available from the corresponding author on reasonable request.

## Abbreviations

ASIA: American Spinal Injury Association
LLA: Lower limb amputation
MEDC: More economically developed country
MESH: Medical subject headings
PRISMA-P: Preferred Reporting Items for Systematic Reviews and Meta-Analysis Protocols
QoL: Quality of Life
SCI: Spinal cord injury
SD: Standard deviation
SF-12: Medical Outcomes Study 12-Item Short-Form Health Survey
SF-36: Medical Outcomes Study 36-Item Short-Form Health Survey
WHOQOL-100: World Health Organization Quality of Life Assessment
WHOQOL-BREF: World Health Organization Quality of Life Assessment-BREF
