# Supplementary material for: Time-related changes in quality of life in persons with lower limb amputation or spinal cord injury: protocol for a systematic review
Source: Syst Rev. 2019 Aug 2;8:191. doi: 10.1186/s13643-019-1108-3 (PMC6676595; doi:10.1186/s13643-019-1108-3)
Supplement: Supplementary file 2 — Search terms. (DOCX 76 kb) [file 13643_2019_1108_MOESM2_ESM.docx]

# Additional Attachment 2: Search Terms:

**Detailed Search Terms:**

**PUBMED:**

"spinal cord injuries"[Mesh] OR spinal cord injury[All Fields] OR "amputation"[Mesh] OR paraplegic[All Fields] OR tetraplegic[All Fields] OR quadriplegic[All Fields] OR Lower Extremity Amputation[All Fields] OR Lower Limb Amputation[All Fields] OR Lower Extremities Amputation[All Fields] OR (Lower[All Fields] AND Limbs Amputation[All Fields]) OR Lower-Extremity Amputation[All Fields] OR Lower-Limb Amputation[All Fields] AND (short form 36[All Fields] OR short form 12[All Fields] OR SF-36[All Fields] OR SF-12[All Fields] OR whoqol[All Fields] OR whoqol bref[All Fields] OR whoqol 100[All Fields]) AND ("humans"[MeSH Terms] AND English[lang] AND "adult"[MeSH Terms])

**EMBASE:**

'spinal cord injury'/exp OR 'spinal cord injury' OR 'leg amputation'/exp OR 'leg amputation' OR 'lower extremity amputation' OR 'lower limb amputation' AND ('short form 36'/exp OR 'short form 36' OR 'short form 12'/exp OR 'short form 12' OR 'whoqol bref questionnaire'/exp OR 'whoqol bref questionnaire' OR 'whoqol bref'/exp OR 'whoqol bref' OR 'whoqol*') AND [english]/lim AND ([adult]/lim OR [aged]/lim)

**Web of Science:**

 (((((("spinal cord injur*" OR "paraplegi*" OR "tetraplegi*" OR "quadriplegi*") OR "lower extremity amputation" OR "lower limb amputation") AND (((("short form 12") OR "short form 36") OR "whoqol*") OR "whoqol bref")))))
